# Sustaining the collaborative chronic care model in outpatient mental health: a matrixed multiple case study

**DOI:** 10.1186/s13012-024-01342-2

**Published:** 2024-02-19

**Authors:** Bo Kim, Jennifer L. Sullivan, Madisen E. Brown, Samantha L. Connolly, Elizabeth G. Spitzer, Hannah M. Bailey, Lauren M. Sippel, Kendra Weaver, Christopher J. Miller

**Affiliations:** 1 Center for Healthcare Organization and Implementation Research (CHOIR), VA Boston Healthcare System, 150 South Huntington Avenue, Boston, MA 02130 USA; 2 Harvard Medical School, 25 Shattuck Street, Boston, MA 02115 USA; 3 Center of Innovation in Long Term Services and Supports (LTSS COIN), VA Providence Healthcare System, 385 Niagara Street, Providence, RI 02907 USA; 4 Brown University School of Public Health, 121 South Main Street, Providence, RI 02903 USA; 5 VA Rocky Mountain Mental Illness Research, Education and Clinical Center (MIRECC), 1700 N Wheeling Street, Aurora, CO 80045 USA; 6 VA Northeast Program Evaluation Center, 950 Campbell Avenue, West Haven, CT 06516 USA; 7 Geisel School of Medicine at Dartmouth, 1 Rope Ferry Road, Hanover, NH 03755 USA; 8 VA Office of Mental Health and Suicide Prevention, 810 Vermont Avenue NW, Washington, DC 20420 USA

**Keywords:** Collaborative care, Implementation, Interdisciplinary care, Mental health, Sustainability

## Abstract

**Background:**

Sustaining evidence-based practices (EBPs) is crucial to ensuring care quality and addressing health disparities. Approaches to identifying factors related to sustainability are critically needed. One such approach is Matrixed Multiple Case Study (MMCS), which identifies factors and their combinations that influence implementation. We applied MMCS to identify factors related to the sustainability of the evidence-based Collaborative Chronic Care Model (CCM) at nine Department of Veterans Affairs (VA) outpatient mental health clinics, 3–4 years after implementation support had concluded.

**Methods:**

We conducted a directed content analysis of 30 provider interviews, using 6 CCM elements and 4 Integrated Promoting Action on Research Implementation in Health Services (i-PARIHS) domains as codes. Based on CCM code summaries, we designated each site as high/medium/low sustainability. We used i-PARIHS code summaries to identify relevant factors for each site, the extent of their presence, and the type of influence they had on sustainability (enabling/neutral/hindering/unclear). We organized these data into a sortable matrix and assessed sustainability-related cross-site trends.

**Results:**

CCM sustainability status was distributed among the sites, with three sites each being high, medium, and low. Twenty-five factors were identified from the i-PARIHS code summaries, of which 3 exhibited strong trends by sustainability status (relevant i-PARIHS domain in square brackets): “Collaborativeness/Teamwork [Recipients],” “Staff/Leadership turnover [Recipients],” and “Having a consistent/strong internal facilitator [Facilitation]” during and after active implementation. At most high-sustainability sites only, (i) “Having a knowledgeable/helpful external facilitator [Facilitation]” was variably present and enabled sustainability when present, while (ii) “Clarity about what CCM comprises [Innovation],” “Interdisciplinary coordination [Recipients],” and “Adequate clinic space for CCM team members [Context]” were somewhat or less present with mixed influences on sustainability.

**Conclusions:**

MMCS revealed that CCM sustainability in VA outpatient mental health clinics may be related most strongly to provider collaboration, knowledge retention during staff/leadership transitions, and availability of skilled internal facilitators. These findings have informed a subsequent CCM implementation trial that prospectively examines whether enhancing the above-mentioned factors within implementation facilitation improves sustainability. MMCS is a systematic approach to multi-site examination that can be used to investigate sustainability-related factors applicable to other EBPs and across multiple contexts.

**Supplementary Information:**

The online version contains supplementary material available at 10.1186/s13012-024-01342-2.

Contributions to the literature

- We examined the ways in which the sustainability of the evidence-based Collaborative Chronic Care Model differed across nine outpatient mental health clinics where it was implemented.
- This work demonstrates a unique application of the Matrixed Multiple Case Study (MMCS) method, originally developed to identify factors and their combinations that influence implementation, to investigate the long-term sustainability of a previously implemented evidence-based practice.
- Contextual influences on sustainability identified through this work, as well as the systematic approach to multi-site examination offered by MMCS, can inform future efforts to sustainably implement and methodically evaluate an evidence-based practice’s uptake and continued use in routine care.

## Background

The sustainability of evidence-based practices (EBPs) over time is crucial to maximize the public health impact of EBPs implemented into routine care. Implementation evaluators focus on sustainability as a central implementation outcome, and funders of implementation efforts seek sustained long-term returns on their investment. Furthermore, practitioners and leadership at implementation sites face the task of sustaining an EBP’s usage even after implementation funding, support, and associated evaluation efforts conclude. The circumstances and influences contributing to EBP sustainability are therefore of high interest to the field of implementation science.

Sustainability depends on the specific EBP being implemented, the individuals undergoing the implementation, the contexts in which the implementation takes place, and the facilitation of (i.e., support for) the implementation. Hence, universal conditions that invariably lead to sustainability are challenging to establish. Even if a set of conditions could be identified as being associated with high sustainability “on average,” its usefulness is questionable when most real-world implementation contexts may deviate from “average” on key implementation-relevant metrics.

Thus, when seeking a better understanding of EBP sustainability, there is a critical need for methods that examine the ways in which sustainability varies in diverse contexts. One such method is Matrixed Multiple Case Study (MMCS) [1], which is beginning to be applied in implementation research to identify factors related to implementation [2–5]. MMCS capitalizes on the many contextual variations and heterogeneous outcomes that are expected when an EBP is implemented across multiple sites. Specifically, MMCS provides a formalized sequence of steps for cross-site analysis by arranging data into an array of matrices, which are sorted and filtered to test for expected factors and identify less expected factors influencing an implementation outcome of interest.

Although the MMCS represents a promising method for systematically exploring the “black box” of the ways in which implementation is more or less successful, it has not yet been applied to investigate the long-term sustainability of implemented EBPs. Therefore, we applied MMCS to identify factors related to the sustainability of the evidence-based Collaborative Chronic Care Model (CCM), previously implemented using implementation facilitation [6–8], at nine VA medical centers’ outpatient general mental health clinics. An earlier interview-based investigation of CCM provider perspectives had identified key determinants of CCM sustainability at the sites, yet characteristics related to the ways in which CCM sustainability differed at the sites are still not well understood. For this reason, our objective was to apply MMCS to examine the interview data to determine factors associated with CCM sustainability at each site.

## Methods

### Clinical and implementation contexts

CCM-based care aims to ensure that patients are treated in a coordinated, patient-centered, and anticipatory manner. This project’s nine outpatient general mental health clinics had participated in a hybrid CCM effectiveness-implementation trial 3 to 4 years prior, which had resulted in improved clinical outcomes that were not universally maintained post-implementation (i.e., after implementation funding and associated evaluation efforts concluded) [7, 9]. This lack of aggregate sustainability across the nine clinics is what prompted the earlier interview-based investigation of CCM provider perspectives that identified key determinants of CCM sustainability at the trial sites [10].

These prior works were conducted in VA outpatient mental health teams, known as Behavioral Health Interdisciplinary Program (BHIP) teams. While there was variability in the exact composition of each BHIP team, all teams consisted of a multidisciplinary set of frontline clinicians (e.g., psychiatrists, psychologists, social workers, nurses) and support staff, serving a panel of about 1000 patients each.

This current project applied MMCS to examine the data from the earlier interviews [10] for the ways in which CCM sustainability differed at the sites and the factors related to sustainability. The project was determined to be non-research by the VA Boston Research and Development Service, and therefore did not require oversight by the Institutional Review Board (IRB). Details regarding the procedures undertaken for the completed hybrid CCM effectiveness-implementation trial, which serves as the context for this project, have been previously published [6, 7]. Similarly, details regarding data collection for the follow-up provider interviews have also been previously published [10]. We provide a brief overview of the steps that we took for data collection and describe the steps that we took for applying MMCS to analyze the interview data. Additional file 1 outlines our use of the Consolidated Criteria for Reporting Qualitative Research (COREQ) Checklist [11].

### Data collection

We recruited 30 outpatient mental health providers across the nine sites that had participated in the CCM implementation trial, including a multidisciplinary mix of mental health leaders and frontline staff. We recruited participants via email, and we obtained verbal informed consent from all participants. Each interview lasted between 30 and 60 min and focused on the degree to which the participant perceived care processes to have remained aligned to the CCM’s six core elements: work role redesign, patient self-management support, provider decision support, clinical information systems, linkages to community resources, and organizational/leadership support [12–14]. Interview questions also inquired about the participant’s perceived barriers and enablers influencing CCM sustainability, as well as about the latest status of CCM-based care practices. Interviews were digitally recorded and professionally transcribed. Additional details regarding data collection have been previously published [10].

### Data analysis

We applied MMCS’ nine analytical steps [1] to the interview data. Each step described below was led by one designated member of the project team, with subsequent review by all project team members to reach a consensus on the examination conducted for each step.

We established the evaluation goal (step 1) to identify the ways in which sustainability differed across the sites and the factors related to sustainability, defining sustainability (step 2) as the continued existence of CCM-aligned care practices—namely, that care processes remained aligned with the six core CCM elements. Table 1 shows examples of care processes that align with each CCM element. As our prior works directly leading up to this project (i.e., design and evaluation of the CCM implementation trial that involved the very sites included in this project [6, 15, 16]) were guided by the Integrated Promoting Action on Research Implementation in Health Services (i-PARIHS) framework [17] and i-PARIHS positions facilitation (the implementation strategy that our trial was testing) as the core ingredient that drives implementation [17], we selected i-PARIHS’ four domains—innovation, recipients, context, and facilitation—as relevant domains under which to examine factors influencing sustainability (step 3). i-PARIHS posits that the successful implementation of an innovation and its sustained use by recipients in a context is enabled by facilitation (both the individuals doing the facilitation and the process used for facilitation). We examined the data on both sustainability and potentially relevant i-PARIHS domains (step 4) by conducting directed content analysis [18] of the recorded and professionally transcribed interview data. We used the six CCM elements and the four i-PARIHS domains as a priori codes.

**Table 1:** Collaborative Chronic Care Model (CCM) sustainability examples per CCM element, adapted from [10, 16, 19]

CCM element	CCM sustainability examples
*Work role redesign:* Providing care that anticipates patients’ needs and preferences through redesigning processes within an interdisciplinary team structure	• Continued involvement of a care coordinator as a specified team member role • Continued running of patient orientation groups as a specified team member task
*Patient self-management support:* Enhancing patients’ self-management skills to help them work toward wellness outside of treatment sessions	• Continued emphasis on the delivery of evidence-based practices • Continued availability of clinic brochures or guidance documents to orient patients to available mental health services
*Provider decision support:* Ensuring that the treatment team’s providers have access to needed clinical expertise	• Continued emphasis on delivery of evidence-based practices (as for the patient self-management support element immediately above) • Continued attention to processes of referral to other clinics
*Clinical information systems:* Using electronic/automated mechanisms to enhance evaluation and coordination of care, with an emphasis on caring for patient populations or panels	• Continued emphasis on patient-level measurement-based care • Continued curation and analysis of aggregated data across the team’s panel of patients
*Linkages to community resources:* Facilitated or systematic relationships with entities outside of the immediate treatment setting to support care delivery and community integration	• Continued development and updated documentation of community linkages • Continued use of linkage procedures that are systematic and available team-wide (rather than idiosyncratic/clinician-specific)
*Organizational / Leadership support:* Providing resources and support to the treatment teams from various levels within the organization, including executive level leaders as well as more direct line supervisors and managers in mental health specialty care services	• Continued emphasis on CCM-based care from mental health leadership • Continued time blocked for team meetings supporting interdisciplinary care

Additional file 2 provides an overview of data input, tasks performed, and analysis output for MMCS steps 5 through 9 described below. We assessed sustainability per site (step 5) by generating CCM code summaries per site, and reached a consensus on whether each site exhibited high, medium, or low sustainability relative to other sites based on the summary data. We assigned a higher sustainability level for sites that exhibited more CCM-aligned care processes, had more participants consistently mention those processes, and considered those processes more as “just the way things are done” at the site. Namely, (i) high sustainability sites had concrete examples of CCM-aligned care processes (such as the ones shown in Table 1) for many of the six CCM elements, which multiple participants mentioned as central to how they deliver care, (ii) low sustainability sites had only a few concrete examples of CCM-aligned care processes, mentioned by only a small subset of participants and/or inconsistently practiced, and (iii) medium sustainability sites matched neither of the high nor low sustainability cases, having several concrete examples of CCM-aligned care process for some of the CCM elements, varying in whether they are mentioned by multiple participants or how consistently they are a part of delivering care. For the CCM code summaries per site, one project team member initially reviewed the coded data to draft the summaries including exemplar quotes. Each summary and relevant exemplar quotes were then reviewed by and refined with input from all six project team members during recurring team meetings to finalize the high, medium, or low sustainability designation to use in the subsequent MMCS steps. Reviewing and refining the summaries for the nine sites took approximately four 60-min meetings of the six project team members, with each site’s CCM code summary taking approximately 20–35 min to discuss and reach consensus on. We referred to lists of specific examples of how the six core CCM elements were operationalized in our CCM implementation trial [19, 20]. Refinements occurred mostly around familiarizing the newer members of the project team (i.e., those who had not participated in our prior CCM-related work) with the examples and definitions. We aligned to established qualitative analysis methods for consensus-reaching discussions [18, 21]. Recognizing the common challenge faced by such discussions in adequately accounting for everyone’s interpretations of the data [22], we drew on Bens’ meeting facilitation techniques [23] that include setting ground rules, ensuring balanced participation from all project team members, and accurately recording decisions and action items.

We then identified influencing factors per site (step 6), by generating i-PARIHS code summaries per site and identifying distinct factors under each domain of i-PARIHS (e.g., *Collaborativeness and teamwork* as a factor under the Recipients domain). For the i-PARIHS code summaries per site, one project team member initially reviewed the coded data to draft the summaries including exemplar quotes. They elaborated on each i-PARIHS domain-specific summary by noting distinct factors that they deemed relevant to the summary, proposing descriptive wording to refer to each factor (e.g., “team members share a commitment to their patients” under the Recipients domain). Each summary, associated factor descriptions, and relevant exemplar quotes were then reviewed and refined with input from all six project team members during recurring team meetings to finalize the relevant factors to use in the subsequent MMCS steps. Finalizing the factors included deciding which similar proposed factor descriptions from different sites to consolidate into one factor and which wording to use to refer to the consolidated factor (e.g., “team members share a commitment to their patients,” “team members collaborate well,” and “team members know each other’s styles and what to expect” were consolidated into the *Collaborativeness and teamwork* factor under the Recipients domain). It took approximately four 60-min meetings of the six project team members to review and refine the summaries and factors for the nine sites, with each site’s i-PARIHS code summary and factors taking approximately 20–35 min to discuss and reach consensus on. We referred to lists of explicit definitions of i-PARIHS constructs that our team members had previously developed and published [16, 24]. We once again aligned to established qualitative analysis methods for consensus-reaching discussions [18, 21], drawing on Bens’ meeting facilitation techniques [23] to adequately account for everyone’s interpretations of the data [22].

We organized the examined data (i.e., the assessed sustainability and identified factors per site) into a sortable matrix (step 7) using Microsoft Excel [25], laid out by influencing factor (row), sustainability (column), and site (sheet). We conducted within-site analysis of the matrixed data (step 8), examining the data on each influencing factor and designating whether the factor (i) was present, somewhat present, or minimally present [based on aggregate reports from the site’s participants; used “minimally present” when, considering all available data from a site regarding a factor, the factor was predominantly weak (e.g., predominantly weak *Ability to continue patient care during COVID* at a medium sustainability site); used “somewhat present” when, considering all available data from a site regarding a factor, the factor was neither predominantly strong nor predominantly weak (e.g., neither predominantly strong nor predominantly weak *Collaborativeness and teamwork* at a low sustainability site)], and (ii) had an enabling, hindering, or neutral/unclear influence on sustainability (designated as “neutral” when, considering all available data from a site regarding a factor, the factor had neither a predominantly enabling nor a predominantly hindering influence on sustainability). These designations of factors’ presence and influence are conceptually representative of what is commonly referred to as magnitude and valence, respectively, by other efforts that construct scoring for qualitative data (e.g., [26, 27]). Like the team-based consensus approach of earlier MMCS steps, factors’ presence and type of influence per site were initially proposed by one project team member after reviewing the matrix’s site-specific data, then refined with input from all project team members during recurring team meetings that reviewed the matrix. Accordingly, similar to the earlier MMCS steps, we aligned to established qualitative methods [18, 21] and meeting facilitation techniques [23] for these consensus-reaching discussions.

We then conducted a cross-site analysis of the matrixed data (step 9), assessing whether factors and their combinations were (i) present across multiple sites, (ii) consistently associated with higher or lower sustainability, and (iii) emphasized at some sites more than others. We noted that any factor may have not come up during interviews with a site because either it is not pertinent or it is pertinent but still did not come up, although we asked an open-ended question at the end of each interview about whether there was anything else that the participant wanted to share regarding sustainability. To adequately account for these possibilities, we decided as a team to regard a factor or a combination of factors as being associated with high/medium/low sustainability if it was identified at a majority (i.e., even if not all) of the sites designated as high/medium/low sustainability (e.g., if the *Collaborativeness and teamwork* factor is identified at a majority, even if not all, of the high sustainability sites, we would find it to be associated with high sustainability). Like the team-based consensus approach of earlier MMCS steps, cross-site patterns were initially proposed by one project team member after reviewing the matrix’s cross-site data, then refined with input from all project team members during recurring team meetings that reviewed the matrix. Accordingly, similar to the earlier MMCS steps, we aligned to established qualitative methods [18, 21] and meeting facilitation techniques [23] for these consensus-reaching discussions. We acknowledged the potential existence of additional factors influencing sustainability that may not have emerged during our interviews and also may vary substantially between sites. For example, adaptation of the CCM, characteristics of the patient population, and availability of continued funding, which are factors that extant literature reports as being relevant to sustainability [28, 29], were not seen in our interview data. To maintain our analytic focus on the factors seen in our data, we did not add these factors to our analysis.

## Results

**Fig. 1:**
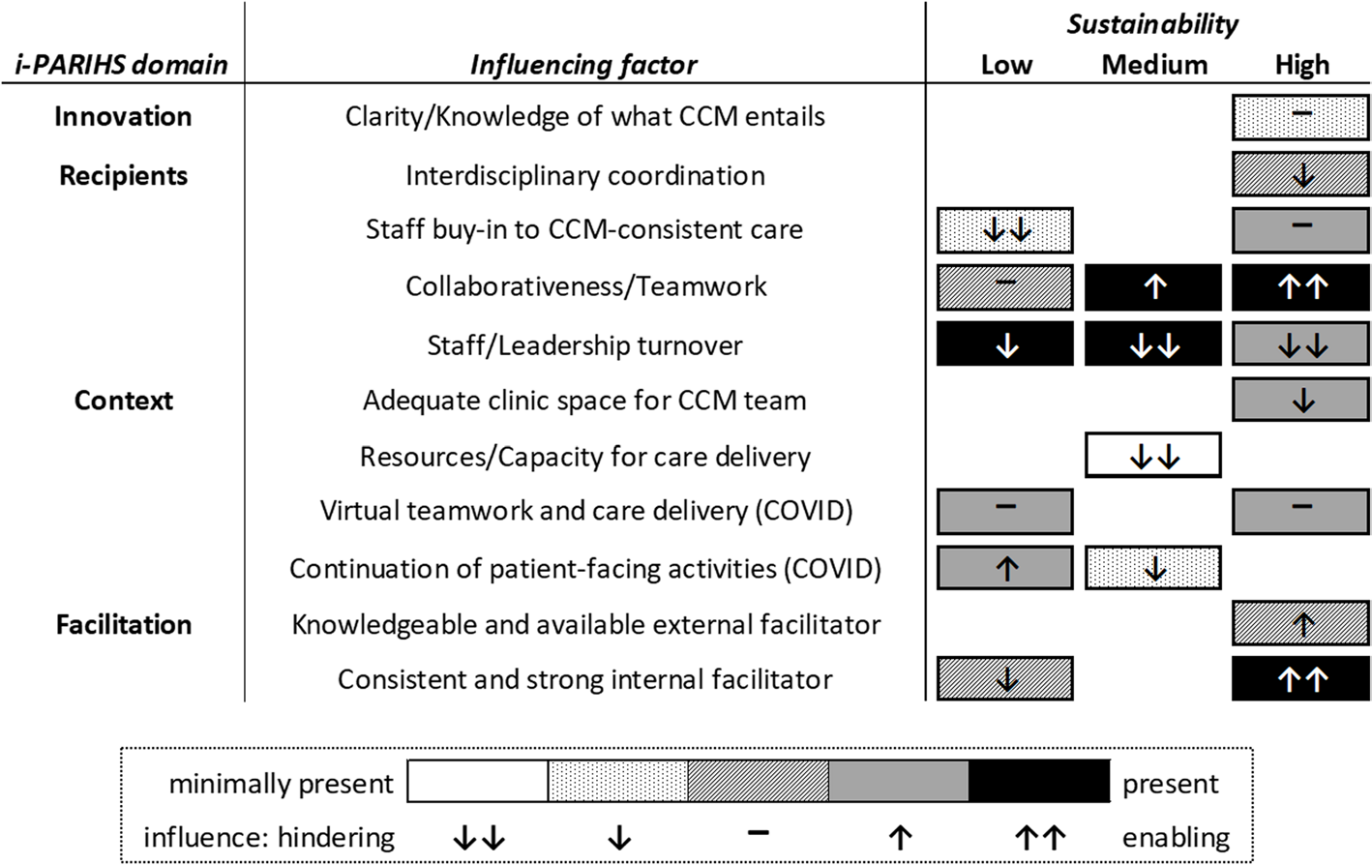
Influencing factors that were identified for at least two sites within a group of high/medium/low sustainability sites

For the nine sites included in this project, we found the degree of CCM sustainability to be split evenly across the sites—three high-, three medium-, and three low-sustainability. Twenty-five total influencing factors were identified under the i-PARIHS domains of Innovation (6), Recipients (6), Context (8), and Facilitation (5). Table 2 shows these identified influencing factors by domain. Figure 1 shows 11 influencing factors that were identified for at least two sites within a group of high/medium/low sustainability sites—e.g., the factor “consistent and strong internal facilitator” is shown as being present at high sustainability sites with an enabling influence on sustainability, because it was identified as such at two or more of the high sustainability sites. Of these 11 influencing factors, four were identified only for sites with high CCM sustainability and two were identified only for sites with medium or low CCM sustainability.

**Table 2:** Factors influencing Collaborative Chronic Care Model (CCM) sustainability, identified under the Integrated Promoting Action on Research Implementation in Health Services (i-PARIHS) domains of Innovation, Recipients, Context, and Facilitation﻿

i-PARIHS domain	Influencing factor
Innovation	• CCM associated with reduced mental health hospitalization
	• CCM provided with staffing/funding support
	• CCM's fit with existing values of care team members
	• Clarity/Knowledge of what CCM entails
	• Incorporation of staff feedback in redesigning care to be CCM-consistent
	• Relative advantage over alternative ways of approaching general mental health care
Recipients	• Collaborativeness/Teamwork
	• Comfortable communication and psychological safety
	• Interdisciplinary coordination
	• Staff buy-in to CCM-consistent care
	• Staff/Leadership turnover
	• Uniformity in how CCM team members approach care-related tasks and/or are skilled
Context	• Virtual teamwork and care delivery (COVID)
	• Continuation of patient-facing activities (COVID)
	• Adequate clinic space for CCM team
	• Overall high workload across clinics
	• Resources/Capacity for care delivery
	• CCM team members subject to significant administrative burden
	• More discussion-based collaboration (rather than relying on consults)
	• Other clinics welcome receiving team’s patients when deemed clinically appropriate by the team
Facilitation	• CCM team members who were not present for the implementation trial are aware of the facilitation
	• Knowledgeable and available external facilitator
	• Consistent and strong internal facilitator
	• Continued internally facilitated CCM team meetings/huddles
	• Having a designated local champion working for culture change

### Key trends in influencing factors associated with high, medium, and/or low CCM sustainability

Three factors across two i-PARIHS domains exhibited strong trends by sustainability status. They were the *Collaborativeness and teamwork* and *Turnover of clinic staff and leadership* factors under the Recipients domain, and the *Having a consistent and strong internal facilitator* factor under the Facilitation domain.

#### Recipients-related factors

*Collaborativeness and teamwork* was present with an enabling influence on CCM sustainability at most high and medium sustainability sites, while it was only somewhat present with a neutral influence on CCM sustainability at most low sustainability sites. When asked what had made their BHIP team work well, a participant from a high sustainability site said,

*“Just a collaborative spirit.”* (Participant 604)

A participant from a medium sustainability site said,

“We joke that [the BHIP teams] are even family, that the teams really do function pretty tightly and they each have their own personality.” (Participant 201)

At the low sustainability sites, willingness to work as a team varied across team members; a participant from a low sustainability site said,

“… I think it has to be the commitment of the people who are on the team. So those that are regularly attending, we get a lot more out of it than those that probably don't ever come [to team meetings].” (Participant 904)

Collaborativeness and teamwork of BHIP team members were often perceived as the highlight of pursuing interdisciplinary care.

*Turnover of clinic staff and leadership* was present with a hindering influence on CCM sustainability at most high, medium, and low sustainability sites.

“We’ve lost a lot of really, really good providers here in the time I’ve been here …,” (Participant 102)

said a participant from a low-sustainability site that had to reconfigure its BHIP teams due to clinic staff shortages. Turnover of mental health clinic leadership made it difficult to maintain CCM practices, especially beyond the teams that participated in the original CCM implementation trial. A participant from a medium sustainability site said,

“Probably about 90 percent of the things that we came up with have fallen by the wayside. Within our team, many of those remain but again, that hand off towards the other teams that I think partly is due to the turnover rate with program managers, supervisors, didn’t get fully implemented.” (Participant 703)

Although turnover was an issue for high sustainability sites as well, there was also indication of the situation improving in recent years; a participant from a high sustainability site said,

“… our attrition rollover rate has dropped quite a bit and I would really attribute that to [the CCM being] more functional and more sustainable and tolerable for the providers.” (Participant 502)

As such, staff and leadership turnover was deemed a major challenge for CCM sustainability for all sites regardless of the overall level of sustainability.

#### Facilitation-related factor

*Having a consistent and strong internal facilitator* was present with an enabling influence on CCM sustainability at high sustainability sites, not identified as an influencing factor at most of the medium sustainability sites, and variably present with a hindering, neutral, or unclear influence on CCM sustainability at low sustainability sites. Participants from a high sustainability site perceived that it was important for the internal facilitator to understand different BHIP team members’ personalities and know the clinic’s history. A participant from another high sustainability site shared that, as an internal facilitator themselves, they focused on recognizing and reinforcing the progress of team members:

“… I'm often the person who kind of [starts] off with, ‘Hey, look at what we've done in this location,’ ‘Hey look at what the team's done this month.’” (Participant 402)

A participant from a low sustainability site had also served as an internal facilitator and recounted the difficulty and importance of readying the BHIP team to function in the long run without their assistance:

“I should have been able to get out sooner, I think, to get it to have them running this themselves. And that was just a really difficult process.” (Participant 301)

Participants, especially from the high and low sustainability sites, attributed their BHIP teams’ successes and challenges to the skills of the internal facilitator.

### Influencing factors identified only for sites with high CCM sustainability

Four factors across four i-PARIHS domains were identified for high sustainability sites and not for medium or low sustainability sites. They were the factors *Details about the CCM being well understood* (Innovation domain), *Interdisciplinary coordination* (Recipients domain), *Having adequate clinic space for CCM team members* (Context domain), and *Having a knowledgeable and helpful external facilitator* (Facilitation domain).

#### Innovation-related factor

*Details about the CCM being well understood* was minimal to somewhat present with an unclear influence on CCM sustainability.

“We’ve … been trying to help our providers see the benefit of team-based care and the episodes-of-care idea, and I would say that is something our folks really have continued to struggle with as well,” (Participant 401)

said a participant from a high sustainability site. “What is considered CCM-based care?” continued to be a question on providers’ minds. A participant from a high sustainability site asked during the interview,

“Is there kind of a clearing house of some of the best practices for [CCM] that you guys have … or some other collection of resources that we could draw from?” (Participant 601)

Although such references are indeed accessible online organization-wide, participants were not always aware of those resources or what exactly CCM entails.

#### Recipients-related factor

*Interdisciplinary coordination* was somewhat present with a hindering, neutral, or unclear influence on CCM sustainability. Coordination between psychotherapy and psychiatry providers was deemed difficult by participants from high-sustainability sites. A participant said,

“We were initially kind of top heavy on the psychiatry so just making sure we have … therapy staff balancing that out [has been important].” (Participant 501)

Another participant perceived that BHIP teams were helpful in managing.

… ﻿‘sibling rivalry’ between different disciplines … because [CCM] puts us all in one team and we communicate.” (Participant 505)

Interdisciplinary coordination was understood by the participants as being necessary for effective CCM-based care yet difficult to achieve.

#### Context-related factor

*Having adequate clinic space for CCM team members* was minimal to somewhat present with a hindering, neutral, or unclear influence on CCM sustainability. COVID-19 led to changes in how clinic space was used/assigned. A participant from a high sustainability site remarked,

“Pre-COVID everything was in a room instead of online. And now all our meetings are online and so it's actually really easy for the supervisors to be able to rotate through them and then, you know, they can answer programmatic questions ….” (Participant 402)

Participants from another high sustainability site found that issues regarding limited clinic space were both exacerbated and alleviated by COVID, with the mental health service losing space to vaccine clinics but more mental health clinicians teleworking and in less need of clinic space. Virtual connections were seen to alleviate some physical workspace-related concerns.

#### Facilitation-related factor

*Having a knowledgeable and helpful external facilitator* was variably present; when present, it had an enabling influence on CCM sustainability. Participants from a high sustainability site noted how many of the external facilitator’s efforts to change the BHIP team’s work processes very much remained over time. An example of a change was to have team meetings be structured to meet evolving patient needs. Team members came to meetings with the shared knowledge and expectation that,

“… we need to touch on folks who are coming out of the hospital, we need to touch on folks with higher acuity needs.” (Participant 402)

Implementation support that sites received from their external facilitator mostly occurred during the time period of the original CCM implementation trial; correspondence with the external facilitator after that trial time period was not common for sites. Participants still largely found the external facilitator to provide helpful guidance and advice on delivering CCM-based care.

### Influencing factors identified only for sites with medium or low CCM sustainability

Two factors were identified for medium or low sustainability sites and not for high sustainability sites. They were the factors *Ability to continue patient care during COVID* and *Adequate resources/capacity for care delivery*. These factors were both under i-PARIHS’ Context domain, unlike the influencing factors above that were identified only for high sustainability sites, which spanned all four i-PARIHS domains.

#### Context-related factors

*Ability to continue patient care during COVID* had a hindering influence on CCM sustainability when minimally present. Participants felt that their CCM work was challenged when delivering care through telehealth was made difficult—e.g., at a medium sustainability site, site policies during the pandemic required a higher number of in-person services than the BHIP team providers expected or desired to deliver. On the other hand, this factor had an enabling influence on CCM sustainability when present. A participant at a low sustainability site mentioned the effect of telehealth on being able to follow up more easily with patients who did not show up for their appointments:

“… my no-show rate has dropped dramatically because if people don’t log on after a couple minutes, I call them. They're like ‘oh, I forgot, let me pop right on,’ whereas, you know, in the face-to-face space, you know, you wait 15 minutes, you call them, it’s too late for them to come in so then they're no shows.” (Participant 102)

The advantages of virtual care delivery, as well as the challenges of getting approvals to pursue it to varying extents, were well recognized by the participants.

*Adequate resources/capacity for care delivery* was minimally present at medium sustainability sites with a hindering influence on CCM sustainability. At a medium sustainability site, although leadership was supportive of CCM, resources were being used to keep clinics operational (especially during COVID) rather than investing in building new CCM-based care delivery processes.

“I think that if my boss came to me, [and asked] what could I do for [the clinics] … I would say even more staff,” (Participant 202)

said a participant from a medium sustainability site. At the same time, the participant, as many others we interviewed, understood and emphasized the need for BHIP teams to proceed with care delivery even when resources were limited:

“… when you’re already dealing with a very busy clinic, short staff and then you’re hit with a pandemic you handle it the best that you can.” (Participant 202)

Participants felt the need for basic resource requirements to be met in order for CCM-based care to be feasible.

## Discussion

In this project, we examined factors influencing the sustainability of CCM-aligned care practices at general mental health clinics within nine VA medical centers that previously participated in a CCM implementation trial. Guided by the core CCM elements and i-PARIHS domains, we conducted and analyzed CCM provider interviews. Using MMCS, we found CCM sustainability to be split evenly across the nine sites (three high, three medium, and three low), and that sustainability may be related most strongly to provider collaboration, knowledge retention during staff/leadership transitions, and availability of skilled internal facilitators.

In comparison to most high sustainability sites, participants from most medium or low sustainability sites did not mention a knowledgeable and helpful external facilitator who enabled sustainability. Participants at the high sustainability sites also emphasized the need for clarity about what CCM-based care comprises, interdisciplinary coordination in delivering CCM-aligned care, and adequate clinic space for BHIP team members to connect and collaborate. In contrast, in comparison to participants at most high sustainability sites, participants at most medium or low sustainability sites emphasized the need for better continuity of patient-facing activities during the COVID-19 pandemic and more resources/capacity for care delivery. A notable difference between these two groups of influencing factors is that the ones emphasized at most high sustainability sites are more CCM-specific (e.g., external facilitator with CCM expertise, knowledge, and structures to support delivery of CCM-aligned care), while the ones emphasized at most medium or low sustainability sites are factors that certainly relate to CCM sustainability but are focused on care delivery operations *beyond* CCM-aligned care (e.g., COVID’s widespread impacts, limited staff availability). In short, an emphasis on immediate, short-term clinical needs in the face of the COVID-19 pandemic and staffing challenges appeared to sap sites’ enthusiasm for sustaining more collaborative, CCM-consistent care processes.

Our previous qualitative analysis of these interview data suggested that in order to achieve sustainability, it is important to establish appropriate infrastructure, organizational readiness, and mental health service- or department-wide coordination for CCM implementation [10]. The findings from the current project augment these previous findings by highlighting the specific factors associated with higher and lower CCM sustainability across the project sites. This additional knowledge provides two important insights into what CCM implementation efforts should prioritize with regard to the previously recommended appropriate infrastructure, readiness, and coordination. First, for knowledge retention and coordination during personnel changes (including any changes in internal facilitators through and following implementation), care processes and their specific procedures should be established and documented in order to bring new personnel up to speed on those care processes. Management sciences, as applied to health care and other fields, suggest that such organizational knowledge retention can be maximized when there are (i) structures set up to formally recognize/praise staff when they share key knowledge, (ii) succession plans to be applied in the event of staff turnover, (iii) opportunities for mentoring and shadowing, and (iv) after action reviews of conducted care processes, which allow staff to learn about and shape the processes themselves [30–33]. Future CCM implementation efforts may thus benefit from enacting these suggestions alongside establishing and documenting CCM-based care processes and associated procedures.

Second, efforts to implement CCM-aligned practices into routine care should account for the extent to which sites’ more fundamental operational needs are met or being addressed. That information can be used to appropriately scope the plan, expectations, and timeline for implementation. For instance, ongoing critical staffing shortages or high turnover [34] at a site are unlikely to be resolved through a few months of CCM implementation. In fact, in that situation, it is possible that CCM implementation efforts could lead to reduced team effectiveness in the short term, given the effort required to establish more collaborative and coordinated care processes [35]. Should CCM implementation move forward at a given site, implementation goals ought to be set on making progress in realms that are within the implementation effort’s control (e.g., designing CCM-aligned practices that take staffing challenges into consideration) [36, 37] rather than on factors outside of the effort’s control (e.g., staffing shortages). As healthcare systems determine how to deploy support (e.g., facilitators) to sites for CCM implementation, they would benefit from considering whether it is primarily CCM expertise that the site needs at the moment, or more foundational organizational resources (e.g., mental health staffing, clinical space, leadership enhancement) [38] to first reach an operational state that can most benefit from CCM implementation efforts at a later point in time. There is growing consensus across the field that the readiness of a healthcare organization to innovate is a prerequisite to successful innovation (e.g., CCM implementation) regardless of the specific innovation [39, 40]. Several promising strategies specifically target these organizational considerations for implementing evidence-based practices (e.g., [41, 42]). Further, recent works have begun to more clearly delineate leadership-related, climate-related, and other contextual factors that contribute to organizations’ innovation readiness [43], which can inform healthcare systems’ future decisions regarding preparatory work leading to, and timing of, CCM implementation at their sites.

These considerations informed by MMCS may have useful implications for implementation strategy selection and tailoring for future CCM implementation efforts, especially in delineating the target level (e.g., system, organizational, clinic, individual) and timeline of implementation strategies to be deployed. For instance, of the three factors found to most notably trend with CCM sustainability, *Collaborativeness and teamwork* may be strengthened through shorter-term team-building interventions at the organizational and/or clinic levels [38], *Turnover of clinic staff and leadership* may be mitigated by aiming for longer-term culture/climate change at the system and/or organizational levels [44–46], and *Having a consistent and strong internal facilitator* may be ensured more immediately by selecting an individual with fitting expertise/characteristics to serve in the role [15] and imparting innovation/facilitation knowledge to them [47]. Which of these factors to focus on, and through what specific strategies, can be decided in partnership with an implementation site—for instance, candidate strategies can be identified based on ones that literature points to for addressing these factors [48], systematic selection of the strategies to move forward can happen with close input from site personnel [49], and explicit further specification of those strategies [50] can also happen in collaboration with site personnel to amply account for site-specific contexts [51].

As is common for implementation projects, the findings of this project are highly context-dependent. It involves the implementation of a specific evidence-based practice (the CCM) using a specific implementation strategy (implementation facilitation) at specific sites (BHIP teams within general mental health clinics at nine VA medical centers). For such context-dependent findings to be transferable [52, 53] to meaningfully inform future implementation efforts, sources of variation in the findings and how the findings were reached must be documented and traceable. This means being explicit about each step and decision that led up to cross-site analysis, as MMCS encourages, so that future implementation efforts can accurately view and consider why and how findings might be transferable to their own work. For instance, beyond the finding that *Turnover of clinic staff and leadership* was a factor present at most of the examined sites, MMCS’ traceable documentation of qualitative data associated with this factor at high sustainability sites also allowed highlighting the perception that CCM implementation is contributing to mitigating turnover of providers in the clinic over time, which may be a crucial piece of information that fuels future CCM implementation efforts.

Furthermore, to compare findings and interpretations across projects, consistent procedures for setting up and conducting these multi-site investigations are indispensable [54–56]. Although many projects involve multiple sites and assess variations across the sites, it is less common to have clearly delineated protocols for conducting such assessments. MMCS is meant to target this very gap, by offering a formalized sequence of steps that prompt specification of analytical procedures and decisions that are often interpretive and left less specified. MMCS uses a concrete data structure (the matrix) to traceably organize information and knowledge gained from a project, and the matrix can accommodate various data sources and conceptual groundings (e.g., guiding theories, models, and frameworks) that may differ from project to project – for instance, although our application of MMCS aligned to i-PARIHS, other projects applying MMCS [2, 5] use different conceptual guides (e.g., Consolidated Framework for Implementation Research [57], Theoretical Domains Framework [58]). Therefore, as more projects align to the MMCS steps [1] to identify factors related to implementation and sustainability, better comparisons, consolidations, and transfers of knowledge between projects may become possible.

This project has several limitations. First, the high, medium, and low sustainability assigned to the sites were based on the sites’ CCM sustainability relative to one another, rather than based on an external metric of sustainability. As measures of sustainability such as the Program Sustainability Assessment Tool [59, 60] and the Sustainment Measurement System Scale [61] become increasingly developed and tested, future projects may consider the feasibility of incorporating such measures to assess each site’s sustainability. In our case, we worked on addressing this limitation by using a consensus approach within our project team to assign sustainability levels to sites, as well as by confirming that the sites that we designated as high sustainability exhibited CCM elements that we had previously observed at the end of their participation in the original CCM implementation trial [19]. Second, we did not assign strict thresholds above/below which the counts or proportions of data regarding a factor would automatically indicate whether the factor (i) was present, somewhat present, or minimally present and (ii) had an enabling, hindering, or neutral/unclear influence on sustainability. This follows widely accepted qualitative analytical guidance that discourages characterizing findings solely based on the frequency with which a notion is mentioned by participants [62–64], in order to prevent unsubstantiated inferences or conclusions. We sought to address this limitation in two ways: We carefully documented the project team’s rationale for each consensus reached, and we reviewed all consensuses reached in their entirety to ensure that any two factors with the same designation (e.g., “minimally present”) do not have associated rationale that conflict across those factors. These endeavors we undertook closely adhere to established case study research methods [65], which MMCS builds on, that emphasize strengthening the validity and reliability of findings through documenting a detailed analytic protocol, as well as reviewing data to ensure that patterns match across analytic units (e.g., factors, interviewees, sites). Third, our findings are based on three sites each for high/medium/low sustainability, and although we identified single factors associated with sustainability, we found no specific combinations of factors’ presence and influence that were repeatedly existent at a majority of the sites designated as high/medium/low sustainability. Examining additional sites on the factors identified through this work (as we will for our subsequent CCM implementation trial described below) will allow more opportunities for repeated combinations and other factors to emerge, making possible firmer conclusions regarding the extent to which the currently identified factors and absence of identified combinations are applicable beyond the sites included in this study. Fourth, the identified influencing factor “leadership support for CCM” (under the Context domain of the i-PARIHS framework) substantially overlaps in concept with the core “organizational/leadership support” element of the CCM. To avoid circular reasoning, we used leadership support-related data to inform our assignment of sites’ high, medium, or low CCM sustainability, rather than as a reason for the sites’ CCM sustainability. In reality, strong leadership support may both result from and contribute to implementation and sustainability [16, 66], and thus causal relationships between the i-PARIHS-aligned influencing factors and the CCM elements (possibly with feedback loops) warrant further examination to most appropriately use leadership support-related data in future analyses of CCM sustainability. Fifth, findings may be subject to both social desirability bias in participants providing more positive than negative evidence of sustainability (especially participants who are responsible for implementing and sustaining CCM-aligned care at their site) and the project team members’ bias in interpreting the findings to align to their expectations of further effort being necessary to sustainably implement the CCM. To help mitigate this challenge, the project interviewers strove to elicit from participants both positive and negative perceptions and experiences related to CCM-based care delivery, both of which were present in the examined interview data.

Future work stemming from this project is twofold. Regarding CCM implementation, we will conduct a subsequent CCM implementation trial involving eight new sites to prospectively examine how implementation facilitation with an enhanced focus on these findings affects CCM sustainability. We started planning for sustainability prior to implementation, looking to this work for indicators of specific modifications needed to the previous way in which we used implementation facilitation to promote the uptake of CCM-based care﻿ [67]. Findings from this work suggest that sustainability may be related most strongly to (i) provider collaboration, (ii) knowledge retention during staff/leadership transitions, and (iii) availability of skilled internal facilitators. Hence, we will accordingly prioritize developing procedures for (i) regular CCM-related information exchange amongst BHIP team members, as well as between the BHIP team and clinic leadership, (ii) both translating knowledge to and keeping knowledge documented at the site, and (iii) supporting the sites’ own personnel to take the lead in driving CCM implementation.

Regarding MMCS, we will continuously refine and improve the method by learning from other projects applying, testing, and critiquing MMCS. Outside of our CCM-related projects, examinations of implementation data using MMCS are actively underway for various implementation efforts including that of a data dashboard for decision support on transitioning psychiatrically stable patients from specialty mental health to primary care [2], a peer-led healthy lifestyle intervention for individuals with serious mental illness [3], screening programs for intimate partner violence [4], and a policy- and organization-based health system strengthening intervention to improve health systems in sub-Saharan Africa [5]. As MMCS is used by more projects that differ from one another in their specific outcome of interest, and especially in light of our MMCS application that examines factors related to sustainability, we are curious whether certain proximal to distal outcomes are more subject to heterogeneity in influencing factors than other outcomes. For instance, sustainability outcomes, which are tracked following a longer passage of time than some other outcomes, may be subject to more contextual variations that occur over time and thus could particularly benefit from being examined using MMCS. We will also explore MMCS’ complementarity with coincidence analysis and other configurational analytical approaches [68] for examining implementation phenomena. We are excited about both the step-by-step traceability that MMCS can bring to such methods and those methods’ computational algorithms that can be beneficial to incorporate into MMCS for projects with larger numbers of sites. For example, Salvati and colleagues [69] described both the inspiration that MMCS provided in structuring their data as well as how they addressed MMCS’ visualization shortcomings through their innovative data matrix heat mapping, which led to their selection of specific factors to include in their subsequent coincidence analysis. Coincidence analysis is an enhancement to qualitative comparative analysis and other configurational analytical methods, in that it is formulated specifically for causal inference [70]. Thus, in considering improved reformulations of MMCS’ steps to better characterize examined factors as explicit causes to the outcomes of interest, we are inspired by and can draw on coincidence analysis’ approach to building and evaluating causal chains that link factors to outcomes. Relatedly, we have begun to actively consider the potential contribution that MMCS can make to hypothesis generation and theory development for implementation science. As efforts to understand the mechanisms through which implementation strategies work are gaining momentum [71–73], there is an increased need for methods that help decompose our understanding of factors that influence the mechanistic pathways from strategies to outcomes [74]. Implementation science is facing the need to develop theories, beyond frameworks, which delineate hypotheses for observed implementation phenomena that can be subsequently tested [75]. The methodical approach that MMCS offers can aid this important endeavor, by enabling data curation and examination of pertinent factors in a consistent way that allows meaningful synthesis of findings across sites and studies. We see these future directions as concrete steps toward elucidating the factors related to sustainable implementation of EBPs, especially leveraging data from projects where the number of sites is much smaller than the number of factors that may matter—which is indeed the case for most implementation projects.

## Conclusions

Using MMCS, we found that provider collaboration, knowledge retention during staff/leadership transitions, and availability of skilled internal facilitators may be most strongly related to CCM sustainability in VA outpatient mental health clinics. Informed by these findings, we have a subsequent CCM implementation trial underway to prospectively test whether increasing the aforementioned factors within implementation facilitation enhances sustainability. The MMCS steps used here for systematic multi-site examination can also be applied to determining sustainability-related factors relevant to various other EBPs and implementation contexts.

### Supplementary Information

**Additional file 1. **COREQ (COnsolidated criteria for REporting Qualitative research) Checklist.

**Additional file 2. **Data input, tasks performed, and analysis output for MMCS Steps 5 through 9.﻿

## Data Availability

The data analyzed during the current project are not publicly available because participant privacy could be compromised.

## Abbreviations

BHIP: Behavioral Health Interdisciplinary Program
CCM: Collaborative Chronic Care Model
COREQ: Consolidated Criteria for Reporting Qualitative Research
COVID: coronavirus disease
EBP: evidence-based practice
IRB: Institutional Review Board
i-PARIHS: Integrated Promoting Action on Research Implementation in Health Services
MMCS: Matrixed Multiple Case Study
VA: United States Department of Veterans Affairs
