# Supplementary material for: Sustaining the collaborative chronic care model in outpatient mental health: a matrixed multiple case study
Source: Implement Sci. 2024 Feb 19;19:16. doi: 10.1186/s13012-024-01342-2 (PMC10875770; doi:10.1186/s13012-024-01342-2)
Supplement: Supplementary file 2 — Additional file 2. Data input, tasks performed, and analysis output for MMCS Steps 5 through 9.﻿ [file 13012_2024_1342_MOESM2_ESM.pdf]

Additional File 2. Data input, tasks performed, and analysis output for MMCS Steps 5 through 9.

MMCS step	*Data input*	*Tasks performed*	*Analysis output*
**5. Assessed sustainability per site**	• CCM-coded interview data	• Generated CCM code summaries per site<br>• Reached consensus on whether each site exhibited high, medium, or low sustainability	• Assignment of high/medium/low sustainability level to each of the 9 sites: 3 high, 3 medium, and 3 low
**6. Identified influencing factors per site**	• i-PARIHS-coded interview data	• Generated i-PARIHS code summaries per site, including descriptions of factors deemed relevant to each summary<br>• Reached consensus on identified factors per site and a consolidated list of identified factors across all the sites	• Designation of relevant factors for each of the 9 sites<br>• List of 25 factors under the i-PARIHS domains of Innovation (6), Recipients (6), Context (8), and Facilitation (5)
**7. Organized the assessed sustainability and identified factors per site into a sortable matrix**	• CCM sustainability assessed per site<br>• i-PARIHS-aligned factors identified per site	• Arranged the data into a matrix using Microsoft Excel: factors on rows, sustainability on columns, and sites on sheets	• Matrix of 25 rows (factors), 3 columns (sustainability), and 9 sheets (sites) that is sortable and filterable
**8. Conducted within-site analysis of the matrixed data**	• Each site-specific sheet of the matrix	• Examined the data on each factor per site<br>• Reached consensus on whether the factor was present, somewhat present, or minimally present at the site<br>• Reached consensus on whether the factor had an enabling, hindering, or neutral/unclear influence on sustainability at the site	• Designation (and insertion into the matrix) of the presence and influence of each of the 25 factors for each of the 9 sites

**9. Conducted cross-site analysis of the matrixed data**	• Matrix with presence and influence specification for each factor per site, with the sheets (sites) ordered by sustainability	• Assessed whether factors and their combinations were (i) present across multiple sites, (ii) consistently associated with higher or lower sustainability, and (iii) emphasized at some sites more than others<br>• Regarded a factor or a combination of factors as being associated with high/medium/low sustainability if it was identified at a majority (i.e., even if not all) of the sites designated as high/medium/low sustainability	• 11 factors (and no combinations of factors' presence and influence) identified for at least 2 sites within a group of high/medium/low sustainability sites, under the i-PARIHS domains of Innovation (1), Recipients (4), Context (4), and Facilitation (2)

CCM: Collaborative Chronic Care Model
i-PARIHS: Integrated Promoting Action on Research Implementation in Health Services
MMCS: Matrixed Multiple Case Study
